# Simulation of Sheared Suspensions With a Parallel Implementation of QDPD

**DOI:** 10.6028/jres.109.017

**Published:** 2004-04-01

**Authors:** James S. Sims, Nicos Martys

**Affiliations:** National Institute of Standards and Technology, Gaithersburg, MD 20899-8911

**Keywords:** dissipative particle dynamics, domain decomposition, mesoscopic modeling, parallel algorithms, rheology, spatial decomposition, suspensions

## Abstract

A parallel quaternion-based dissipative particle dynamics (QDPD) program has been developed in Fortran to study the flow properties of complex fluids subject to shear. The parallelization allows for simulations of greater size and complexity and is accomplished with a parallel link-cell spatial (domain) decomposition using MPI. The technique has novel features arising from the DPD formalism, the use of rigid body inclusions spread across processors, and a sheared boundary condition. A detailed discussion of our implementation is presented, along with results on two distributed memory architectures. A parallel speedup of 24.19 was obtained for a benchmark calculation on 27 processors of a distributed memory cluster.

## 1. Introduction

Understanding the flow properties of complex fluids like suspensions (e.g., colloids, ceramic slurries, and concrete) is of importance to industry and presents a significant theoretical challenge. The computational modeling of such systems is also a great challenge because it is difficult to track boundaries between different fluid/fluid and fluid/solid phases. Recently, a new computational method called dissipative particle dynamics (DPD) [2] has been introduced which has several advantages over traditional computational dynamics methods while naturally accommodating such boundary conditions. In structure, a DPD algorithm looks much like molecular dynamics (MD), where atomistic particles move according to Newton’s laws. However, the DPD “particles” are a mesoscopic description of the fluid, and do not represent individual atoms or molecules, but loosely correspond to “lumps” of fluid or clusters of molecules. As a result, the interactions between the DPD particles are not directly based on a Lennard-Jones potential, but are typically subject to three types of forces, namely, conservative forces, dissipative forces, and a random force. All of the forces conserve momentum and mass. The conservative force is simply a central force, derivable from some potential. The dissipative force is proportional to the difference in velocity between particles and acts to slow down their relative motion. The dissipative force can be shown to produce a viscous effect. The random force (usually based on a Gaussian random noise) helps maintain the temperature of the system while producing a viscous effect. It can be shown that, in order to maintain a well defined temperature by way of consistency with a fluctuation-dissipation theorem [3], coefficients describing the strength of the dissipative and random forces must be coupled. By mapping of the DPD equations of motion to the Fokker-Planck equation [4], it has been demonstrated that the DPD equations can recover hydrodynamic behavior consistent with the Navier-Stokes equations.

As in MD, the forces on each particle are computed in each time step. The particles are then moved and the forces recomputed. In DPD the interparticle interactions are chosen to allow for much larger time steps so that physical behavior, on time scales many orders of magnitude greater than that possible with MD, may be studied. The original DPD algorithm [2] used an Euler algorithm for updating the positions of the free particles (which represent “lumps” of fluids), and a leap frog algorithm for updating the positions of solid inclusions (rigid bodies). Our algorithm QDPD [5], for quarternion-based dissipative particle dynamics, is a modification of DPD that uses the velocity-Verlet algorithm of Groot and Warren [6] to update the positions of both the free particles and the solid inclusions. The velocity-Verlet algorithm for DPD [5] is chosen because it is less sensitive to variation in time step size than the Euler algorithm. The solid inclusion motion is determined from the quaternion-based scheme of Omelayan [7] (hence the Q in QDPD).

QDPD in its present form is being used to study the steady-shear viscosity of a suspension of solid inclusions (such as ellipsoids) in a Newtonian fluid. The model consists of *N* particles moving in a continuum domain of volume *V*. As in MD the system is completely defined by specifying all *N* positions ***r****_i_* and momenta ***p****_i_* (*i* = 1, …, *N*). To model a rigid body inclusion in a fluid, a subset of the DPD particles are initially assigned a location in space so that they approximate the shape of the object [8]. The motion of these particles is then constrained so that their relative positions never change. The total force and torque are determined from the DPD particle interactions and the rigid body moves according to the Euler equations. As mentioned above, our simulations use a quaternion-based scheme developed by Omelayan and modified by Martys and Mountain [5] for a velocity-Verlet algorithm to integrate the equations of motion. Finally, we use a Lees-Edwards boundary condition [9] (pp 246–247) to produce a shearing effect akin to an applied strain at the boundaries.

**Fig. 1:**
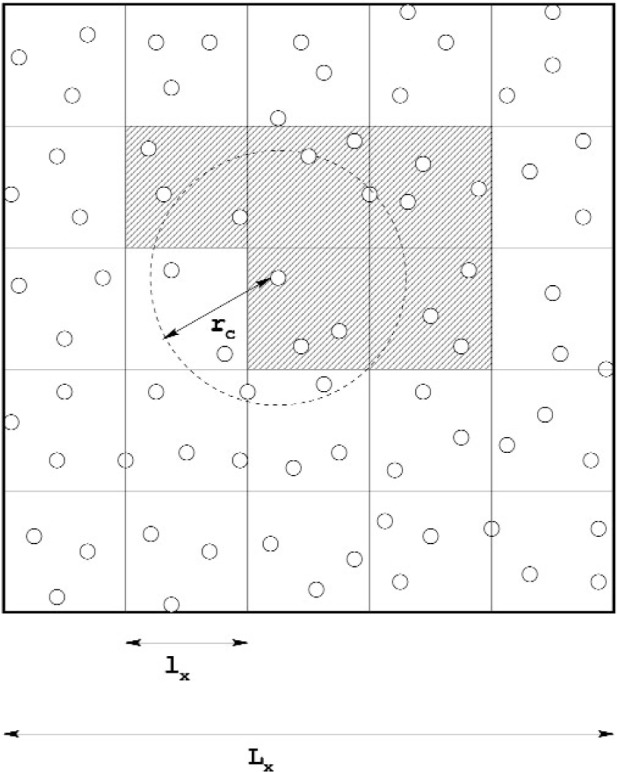
Schematic diagram of link-cell algorithm for a two dimensional system (after Tildesley, Pinches, and Smith [11]).

**Fig. 2:**
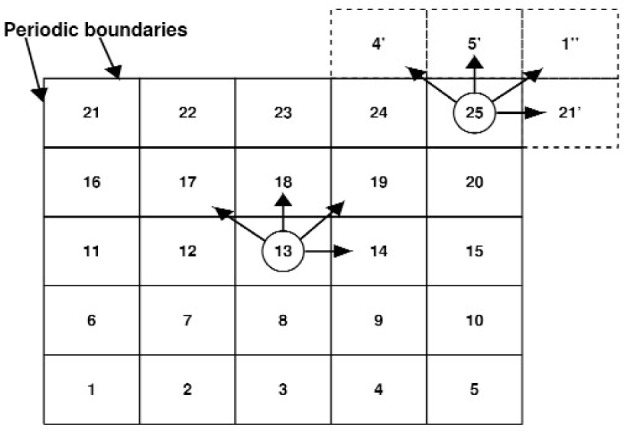
Schematic 2-D representation of the link-cell algorithm.

The basic idea is to compute all of the forces on each particle (which accounts for the momenta change in the collision phase) during each time step, and then move the particles (propagation phase). The forces are short-range and are a sum of contributions over pairs of particles. The interaction decays rapidly with separation, which means that only particles closer than some *cutoff* distance *r*_c_ need be considered. Several methods are available for identifying the nearest neighbors of a particle, i.e, those within the cutoff distance. QDPD uses an implementation of the link-cell method of Quentrec et al. [10] described in Allen and Tildesley’s book [9] (pp. 149–152). Here, the simulation box is partitioned into a number of cells. For example, see Fig. 1, which depicts a 2D system. To find the particles within the cutoff distance *r*_c_ of the particle shown in the central cell, it is sufficient to *only consider* particles within the central cell and each of its eight nearest neighbor cells (where *r*_c_ is ≤ the cell widths in *X* and *Y*, *l_x_* and *l_y_*). The use of Newton’s third law makes it possible for us to *only* have to consider half of the nearest neighboring cells, which are cross-hatched (lines parallel to the right-diagonal in the cell) in the figure. Generalizing this to all particles in the system, a linked list of all the particles contained in each cell is constructed every timestep. Then, for each particle, the selection of all *particles* within the cutoff is achieved by looping over one half (considering Newton’s third law) of all nearest neighbor cells, and considering *only* the particles within these cells. We show this schematically in Fig. 2.

The (forces calculation) search scheme involves an outer loop over all 25 link-cells. In this outer loop, each particle in a link-cell interacts with all particles within its link-cell that are within *r*_c_ of the particle. Then there is an inner loop over four of the eight nearest neighbor link-cells, and each particle interacts with all of the particles within the chosen neighbor link-cells that are within *r*_c_ of the particle. For example, particles in cell 13 interact with other particles in 13 plus particles in 17, 18, 19, and 14 that are within the cutoff distance of the chosen particle. Note that to account for the forces on particles in edge cells, periodic boundaries are used to have, for example, 25 interacting with the appropriate nearest neighbor periodic cells 4*′*, 5*′*, 1*″*, and 21*′* (more on this later). The program for figuring out nearest neighbor cells is easy to set up. Introducing cell indices *I_x_* and *I_y_* for the 2D grid in Fig. 2, each cell’s index in the 2D grid can be computed from

$$ICELL(I_{x},I_{y})=1+MOD(I_{x}-1+M_{x}M_{y},M_{x})+MOD(I_{y}-1+M_{x}M_{y},M_{y})M_{x},$$ (1)

where *MOD* is the function which returns the modulo of its arguments and *M_x_* and *M_y_* are the number of cells in *X* and *Y* (*I_x_* = {1, *M_x_*}, *I_y_* = {1, *M_y_*}).

For each cell, one-half of the nearest neighbor cells are given by

$$ICELL(I_{x}+1,I_{y})+ICELL(I_{x}+1,I_{y}+1)+ICELL(I_{x},I_{y}+1)+ICELL(I_{x}-1,I_{y}+1),$$ (2)

which correctly gives the cell neighbors of 13 to be 17, 18, 19, and 14. Now we can explain the treatment of Newton’s third law. A particle in cell 13 interacts with the particles in 8 neighboring cells, but the algorithm only checks particles in 17, 18, 19, and 14. Interactions of particles in 13 with particles in 12, for example, are treated when particles in cell 12 are the focus of attention, and similarly for 7, 8, and 9. Note that this formula also gives the nearest neighbors of particles in cell 25 to be those in 4, 5, 1, and 21 (not the periodic cells 4*′*, 5*′*, 1*″*, and 21*′*). This will be explained later. Because of their regular arrangement, the list of neighboring cells is fixed and may be precomputed once and for all at the beginning of the program (in subroutine MAPS).

## 2. Sequential Link-Cell Algorithm

Incorporating a link-cell search into the velocity-Verlet algorithm gives, in outline,


Read in initial data.
Read in configurational data (solid inclusions).
Set up the map to find neighboring cells (subroutine MAPS).
Perform the QDPD cycle for each time step.


Given the forces ***f***_1_*_i_* acting on particles at time *t*, the fundamental QDPD cycle, repeated for as many timesteps as are in a simulation, is


Compute the new particle positions from


$$r_{2i}=r_{1i}+v_{1i}\Delta t+f_{1i}\Delta t^{2}/2.$$ (3)


Compute the midpoint velocity (velocity at the midpoint of the time step) from


$${\tilde{v}}_{i}=v_{1i}+f_{1i}\Delta t/2.$$ (4)


Create the linked list (subroutines TOPMAP and LINKS).


Calculate new forces ***f***_2_*_i_*.


Compute new velocities from the new forces


$$v_{2i}={\tilde{v}}_{i}+f_{2i}\Delta t/2.$$ (5)

And then the cycle begins again.

In the basic QDPD (MD) cycle above, the ***r***_2_s may belong to particles which have moved out of the simulation box, such as particles in the periodic image of cell 21 represented by the dashed box 21*′* in Fig. 2. This can be handled by introducing another set of coordinates, ***r***_3_, given by

$$\begin{array}{l} r3x(i)=r2x(i)-ANINT(r2x(i)/L_{x})L_{x}\\ r3y(i)=r2y(i)-ANINT(r2y(i)/L_{y})L_{y}\\ r3z(i)=r2z(i)-ANINT(r2z(i)/L_{z})L_{z}, \end{array}$$ (6)

where *L_x_*, *L_y_*, and *L_z_* are the simulation dimensions and ANINT is the function which returns the nearest whole integer to its argument. The ***r***_3_ coordinates are used in creating linked lists of particles in cells prior to the force calculations. Consequently, particles which have moved into 21*′* will end up assigned to 21 which is where the formula for nearest neighbors expects to find them. Hence the ***r***_3_ coordinates make sure that particles are assigned to one of the cells in the QDPD simulation box (i.e., particles are kept within the QDPD simulation box running from (–*L_x_*/2, –*L_y_*/2, –*L_z_*/2) to (*L_x_*/2, *L_y_*/2, *L_z_*/2) in ***r***_3_ space). They also are consistent with the proper mapping of nearest neighbor cells given by the *ICELL*(*I_x_*, *I_y_*) formula. One other point has to do with calculating forces on particles > *r*_c_ away (since the particles may have moved out of the simulation box). In calculating forces, ***r***_2_ coordinates are used, and these ***r***_2_ coordinates may be > *r*_c_ away, a violation of the minimum image condition. To correct for this, the difference between particles in the forces calculation is

$$\begin{array}{l} \Delta r_{2x}(ij)=r2x(i)-r2x(j)-ANINT[(r2x(i)-r2x(j)]/L_{x})L_{x}\\ \Delta r_{2y}(ij)=r2y(i)-r2y(j)-ANINT[(r2y(i)-r2y(j)]/L_{y})L_{y}\\ \Delta r_{2z}(ij)=r2z(i)-r2z(j)-ANINT[(r2z(i)-r2z(j)]/L_{z})L_{z} \end{array}$$ (7)

Consider Fig. 2 again. With the *ANINT* corrections above, particles in the cells 4, 5, 1, and 21 are within *r*_c_ away from particles in cell 25. This is the way our sequential version of the program was written. Our parallel version of the program does this differently. In treating edge cells, a “ghost” layer of cells is added to the QDPD simulation box. The dashed cells in Fig. 2, the nearest neighbor periodic cells, are part of the “ghost” layer of cells. The formation of these “ghost” cells will be discussed in the next section.

**Fig. 3:**
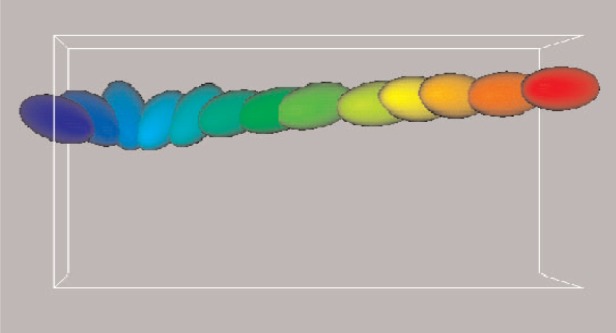
Tumbling of a single ellipsoidal inclusion under shear. Details of the algorithm for a rigid body are given in [5].

**Fig. 4:**
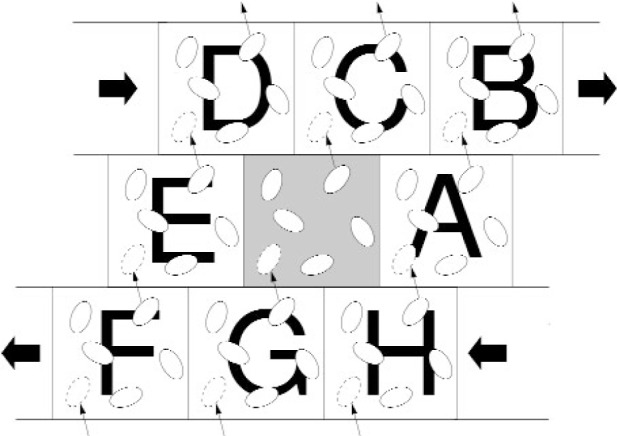
Lees-Edwards boundary conditions for homogeneous shear (adopted from Allen and Tildesley, Computer Simulation of Liquids, Oxford, 1987, Fig. 8.2).

**Fig. 5:**
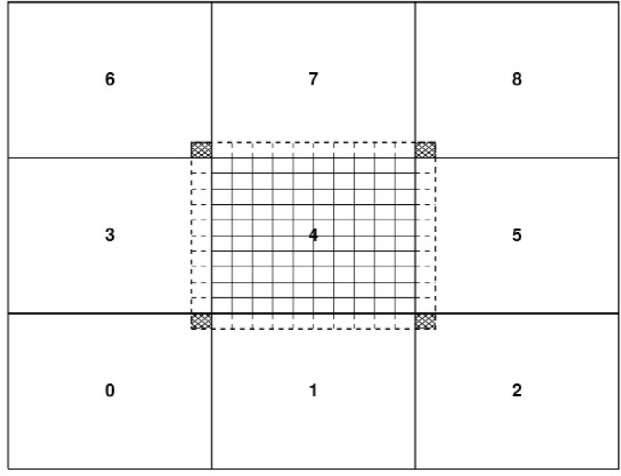
A 9 processor 2-D domain. The small rectangles are cells associated with the link-cell algorithm. The dashed lines correspond to the ghost cells.

In the sequential version of the program, MAPS considers all cells in all layers except for the top layer (the topmost layer in Y in a 3D simulation), and computes only half of these cells, taking into account Newton’s third law. Because QDPD in its present form is being used to study the steady-shear viscosity of a suspension of solid inclusions in a Newtonian fluid, there is a shear boundary condition at the topmost layer of the QDPD simulation box, implemented with the Lees-Edwards boundary conditions [9] (pp. 246–247). These boundary conditions simulate a uniform shear in the *XY* plane (i.e, a constant velocity gradient is set up in the *Y* direction and the actual shear occurs in the *X* direction). Figure 3 shows a time series of the motion of a single ellipsoidal inclusion subject to shear. Proceeding from left to right, the different colors (or greyscale levels) [12] correspond to the time sequence. The single ellipsoid rotation is a well known phenomenon seen in experiments called Jeffery’s orbits. The shearing boundary conditions were obtained by applying a constant strain rate to the right at the top of the figure and to the left at the bottom of the figure. Figure 4 [9] (p. 246) demonstrates this situation in the context of the computer simulation. The central box in the figure is the QDPD simulation box (the entire box in Fig. 5, not just the one on the central processor 4). Boxes in the layer above are moving at a certain speed in the positive direction, and boxes in the layer below are moving at the same speed in the negative direction. To implement this shear boundary condition at the topmost layer (because of Newton’s third law, we only have to treat the top), the top layer is tackled separately in subroutine TOPMAP in our sequential version of the program. Its purpose is to create the list of neighboring cells for the topmost layer of cells, taking into account the movement of the cells with respect to each other due to shear. TOPMAP is called every timestep in the simulation just before the force calculation, but only on the topmost processors. The periodic minimum image convention must also be modified to account for this shear. The ***r***_3_s are modified to be

$$\begin{array}{l} cory=ANINT\left(r2y(i)/L_{y}\right)\\ r3x(i)=r2x(i)-cory*strain\\ r3x(i)=r3x(i)-ANINT\left(r2x(i)/L_{x}\right)L_{x}\\ r3y(i)=r2y(i)-ANINT\left(r2y(i)/L_{y}\right)L_{y}\\ r3z(i)=r2z(i)-ANINT\left(r2z(i)/L_{z}\right)L_{z}, \end{array}$$ (8)

where the upper layer (BCD in Fig. 4) is displaced relative to the central box by an amount *strain*. Similar corrections are made in forces.

## 3. Spatial Decomposition Theory

QDPD was originally written in Fortran 77 as a serial program. A lot of the formalism of the sequential link-cell algorithm relies heavily on the Allen and Tildesley book [9] and computer routines (such as MAPS and TOPMAP) discussed in the book and available on the Web [13]. We have retained the names of the Allen and Tildesley routines in our program and in the discussion. Routines discussed later in the text, such as EXTVOL, LEBC, and MOVPAR, are parallel routines and have no counterpart in Allen and Tildesley. To improve computational performance, a parallelization was done relatively quickly using a simplified version of the replicated data approach and the standard message passing interface library (MPI [1]), as described in Sims et. al. [14]. We reported speedups of as much as 17.5 times on 24 processors of a 32 processor shared memory SGI Origin 2000^1^. When doing a calculation on multiple processors, the total run time can be represented as the sum of computation (cpu) time and communication time, viz.,

$$t=t_{\text{cpu}}+t_{\text{comm}}.$$ (9)

In the replicated data approach, as *P* (number of processors) increases, *t*_cpu_ goes down, but we still have to communicate the same amount of information (proportional to *N* (number of particles), so it doesn’t scale). Also distributed memory machines often do not possess enough memory on a processing node to hold all of the data for a large job. When the goal is to simulate an extremely large system on a distributed-memory computer to allow for the larger total memory of the distributed-memory computer and also to take advantage of a larger number of processors, a different approach is needed. Since the link-cell algorithm we used in the sequential and replicated data approaches breaks the simulation space into domains, it seems natural to map this geometrical, or domain, decomposition onto separate processors. Doing so is the essence of the parallel link-cell technique [11,15]^2^. By subdividing the physical volume among processors, most of the computation becomes local and the communication is minimized so there is, in principle, an *N* / *P* scaling (*N* = number of particles, *P* = number of processors), an efficient approach for distributed-memory computers and networks of workstations.

The basic idea is this:

Split the total volume into *P* domains, where *P* is the number of processors. If we choose a 1D decomposition (“slices of bread”), then the *p*th processor is responsible for particles whose *x*-coordinates lie in the range

$$(p-1)L_{x}/P\leq x<pL_{x}/P.$$ (10)

Similar equations apply for 2D and 3D decompositions for simulation dimensions *L_y_* and *L_z_*. Whether the decomposition is 1D, 2D, or 3D depends on the number of processors. An algorithm due to Plimpton [17] is used to assign *P* processors to a 3D box so as to minimize the surface area (and hence, yield a good load balancing). For *P* processors and a given simulation box of dimensions *L_x_*, *L_y_*, and *L_z_*, the algorithm is the following. Loop through all factorizations of *P* into *P_x_*, *P_y_*, and *P_z_* processors, computing the area of the resulting box, and pick the one with the minimum surface area. Of multiple equal surface areas (for example, *P_x_*, *P_y_*, *P_z_* = (4,2,2), (2,4,2), (2,2,4)), pick the one with *P_x_* ≤ *P_y_* ≤ *P_z_*.

Each processor runs a link-cell program corresponding to a particular domain of the simulation box. For example, in Fig. 5 nine processors were used to divide the 2D simulation space into domains, each processor being assigned to one of the nine domains (here we assign each processor an index, where the indices start at 0). We also show the central processor’s domain being subdivided into cells. To complete the force calculation on particles in cells at the interface between processors, each processor needs to know information about the particles in the adjacent cells, which now will be found on a neighboring processor. To handle this problem we construct an extra layer of cells on each processor at the interface between processors. At each timestep we communicate information across the interface between adjacent processors describing the particles in these edge cells (subroutine EXTVOL). The information that has to be passed by EXTVOL is the information needed for the forces calculations, which is, $r_{3i},r_{2i},{\tilde{v}}_{i}$, and the unique particle number discussed below. For example, in Fig. 5 we show processors 3, 4, and 5 and we also show, with dashed lines, the cells in processors 3 and 5 which are adjacent to 4. Information about the particles in these dashed line cells is communicated to 4, making up “ghost” cells on 4. To complete the “extended volume” needed on processor 4 to compute the forces on all the particles it “owns”, information is communicated (swapped) across the interface between adjacent processors in the *Y* direction as well. To account for the cross-hatched corner cells, the swap in *Y* includes information about not only particles that the processor owns but also information about “other” particles in “ghost” cells. So processor 7 sends information about particles in the dashed line cells as well as the cross-hatched cells (obtained from processors 6 and 8) to processor 4. At this point processor 4 has all the information it needs to calculate forces on all the particles it owns (processor 4 now has information about all the particles shown in the extended volume comprised of the domain of processor 4 plus the surrounding dashed line ghost cells), and similarly all of the other processors have all the information they need. These exchanges of data can be achieved by one set of communications between the processors. A processor only has to communicate once with all of its neighbors, so each processor communicates with at most four other processors (six in 3D), rather than, say 64 in a 64 processor replicated data calculation. Now on each processor, form a link-cell list of all particles in the original volume plus the extended volume. Loop over the particles in the original volume, calculating the forces on them and their pair particle (for conservation of momentum). Care must be taken to add these pair particle forces on particles in the extended volume to the forces on the pair particles in the processor “owning” them, which necessitates an extra set of communications between processors (the reverse of the communication swaps setting up the “ghost” cells). This extra communication step is necessary in the QDPD method since the interparticle force calculation involves the use of a random number for thermal effects and momentum conservation requires that the same random number be used in the equal and opposite force calculation. Finally calculate the new positions of all particles and move the particles which have left a processor to their new home processor. If particles move into domains controlled by other processors, information about the particle (the particle’s properties) must be moved to its new “home” processor. Again these exchanges of data can be achieved by one set of communications between the processors, and are implemented in subroutine MOVPAR. In this set of communications, *all* information about a particle needed for one time step must be communicated, not just the information needed for the forces calculation (the information communicated in EXTVOL as explained above).

Our spatial decomposition program has the following added features. First, following Plimpton [17], we distinguish between “owned” particles and “other” particles, those particles that are on neighboring processors and are part of the extended volume on any given processor. For “other” particles, only the information needed to calculate forces is communicated to neighboring processors. Second, the QDPD technique is being applied to suspensions, so there are two types of particles, “free” particles and particles belonging to ellipsoids (the solid inclusions). A novel feature of this work is that we explicitly do *not* keep all particles belonging to the same ellipsoid on the same processor. Since the largest ellipsoid that might be built can consist of as much as 50 % of all particles, that would be difficult if not impossible to handle without serious load-balancing implications. What we do is assign each particle a unique particle number when it is read in. Each processor has the list of ellipsoid definitions consisting of lists of particles defined by these unique particle numbers. Each processor computes solid inclusion properties for each particle it “owns”, and these properties are globally summed (using MPI_REDUCE [1,18] over all processors so that all processors have the same solid inclusion properties. Since there are only a small number of ellipsoids (relative to the number of particles), the amount of communication necessary for the global sums is small and the amount of extra memory is also relatively small. Hence it is an efficient technique.

## 4. Spatial Decomposition Program Details

After various preliminaries, the program reads information about the simulation space and then calls DOMAIN to figure out the spatial (domain) decomposition. To determine which processors control adjacent domains we identify each processor uniquely by considering each processor in the network as a cell in a link-cell structure. We then use the link-cell algorithm to determine the addresses of a processor’s neighbors. Particles are then mapped onto processors on the basis of their *x*, *y*, and *z* coordinates. For 3D, we denote the number of processors allocated in the *X*, *Y*, and *Z* dimensions by *P_x_*, *P_y_*, and *P_z_*, respectively, so

$$P=P_{x}P_{y}P_{z}.$$ (11)

For a particle at position ***r****_i_* = (*x_i_*, *y_i_*, *z_i_*) in a simulation box with sides of length *L_x_*, *L_y_*, and *L_z_*, with 0 ≤ *x_i_* < *L_x_*, 0 ≤ *y_i_* < *L_x_*, 0 ≤ *z_i_* < *L_z_* the processor coordinates are given by

$$\begin{array}{l} I_{x_{i}}=INT(x_{i}P_{x}/L_{x})\\ I_{y_{i}}=INT(y_{i}P_{y}/L_{y})\\ I_{z_{i}}=INT(z_{i}P_{z}/L_{z}), \end{array}$$ (12)

where *INT* is the function returning the integer part of the argument in brackets. The mapping from processor coordinates $\left(I_{x_{i}},I_{y_{i}},I_{z_{i}}\right)$ to processor index is given by

$$I_{i}=I_{x_{i}}+I_{z_{i}}P_{x}+I_{y_{i}}P_{x}P_{z}.$$ (13)

Coordinates of the center of each processor’s simulation box can be calculated from

$$\begin{array}{l} rorigin(1)=L_{x}((I_{x_{i}}+0.5)/P_{x}-0.5)\\ rorigin(2)=L_{y}((I_{y_{i}}+0.5)/P_{y}-0.5)\\ rorigin(3)=L_{z}((I_{z_{i}}+0.5)/P_{z}-0.5). \end{array}$$ (14)

Particles are allocated to processors on the basis of *I_i_* at the start (subroutines INITPR (for particles) and INITSNEW (for ellipsoids)) and whenever particles are moved. While figuring out the domain decomposition, a processor’s north (+*y* direction), south (−*y* direction), east (+*x* direction), west (−*x* direction), up (+z direction), and down (−*z* direction) neighboring processors are tabulated.

The simulation box size for each processor is given by

$$\begin{array}{l} rprosl(1)=L_{x}/P_{x}\\ rprosl(2)=L_{y}/P_{y}\\ rprosl(3)=L_{z}/P_{z}. \end{array}$$ (15)

In the domain decomposition molecular dynamics cycle (subroutine CYCLE), we now have, on each processor,


Compute the new particle positions from


$$r_{2i}=r_{1i}+v_{1i}\Delta t+f_{1i}\Delta t^{2}/2.$$ (16)


Compute the midpoint velocity (velocity at the midpoint of the time step) from


$${\tilde{v}}_{i}=v_{1i}+f_{1i}\Delta t/2.$$ (17)


Calculate r3s to make sure particles remain in the QDPD box.
Move particles (MOVPAR) to their new home processor based on r3s.
Construct an extended volume consisting of owned cells plus ghost cells (EXTVOL) based on r3s.
EXTVOL calls a subroutine (LEBC) to apply Lees-Edwards shear boundary conditions.
Construct the link-cell list (LINKS) based on r3 coordinates.
Calculate new forces (FORCES), including a call to THIRDLAW, which transfers pair forces back to their home processor and adds them to forces there.
Compute new velocities from the new forces


$$v_{2i}={\tilde{v}}_{i}+f_{2i}\Delta t/2.$$ (18)

The way the Newton’s third law forces are handled in spatial (domain) decomposition is the following. A table is kept of edge particles that are sent in all directions. Then after forces are calculated, THIRDLAW loops over just the “other” particles looking for force contributions that have to be sent back to the processor that “owns” the particle and added to the forces there. THIRDLAW then communicates these Newton’s third law force additions back to the “home” processors of the “other” particles and adds them to the forces there.

Some of these steps require additional explanation. In MOVPAR ***r***_3_*_i_* coordinates are transformed, by subtracting the coordinates of the center of each processor’s simulation box, so that they are in the range

−rprosl(k)/2≤r3(k,i)−rorigin(k)<rprosl(k)/2, (19)

where *rprosl*(*k*) is the size of that processor’s domain in the *k* direction. Particles which don’t meet this criterion have moved out of the processor and are sent to their new home processor. A subtle point is that this is relatively slow motion so we know that the move is to the nearest neighbor in the *k* dimension, the one in the negative or positive direction, depending on whether *r*3(*k*, *i*) − *rorigin*(*k*) < −*rprosl*(*k*) or *r*3(*k*, *i*) − *rorigin*(*k*) ≥ *rprosl*(*k*). This is true for *k* = 2 or 3, but because of the shear boundary condition at the topmost layer, particles may have moved more than one processor away in *X* in a single time step. We handle this by finding the maximum number of swaps in *X* on each processor, then do a global MAX of the values of each processor to determine how many swaps to do.

Next comes the formation of extended volumes using “ghost” cells in EXTVOL. To accomodate the “ghost” cells, the number of cells in each direction is increased by 2. So, for example, for a division of the central processor into 100 cells as in Fig. 5, the *X* and *Y* cell dimensions are 10. *M_x_* and *M_y_*, the cell *X* and *Y* cell dimensions for this processor are 12 (10 + 2) to accomodate the left and right ghost cells. We use the following to define a cell index for particle *i* (*ICELL_i_*)

$$ICELL_{i}=I_{x_{i}}+(I_{y_{i}}-1)M_{x}+(I_{z_{i}}-1)M_{x}M_{y}$$ (20)

where $I_{x_{i}}$, $I_{y_{i}}$, and $I_{z_{i}}$ are now given by

$$\begin{array}{l} I_{x_{i}}=1+INT((r(1,i)S_{x}+0.5)M_{x})\\ I_{y_{i}}=1+INT((r(2,i)S_{y}+0.5)M_{y})\\ I_{z_{i}}=1+INT((r(3,i)S_{z}+0.5)M_{z}). \end{array}$$ (21)

*S_x_*, *S_y_*, and *S_z_* are scale factors whose purpose is to transform coordinates so that a processor’s “own” particles in a domain will have values in the range

$$\begin{array}{l} 2\leq I_{x_{i}}\leq M_{x}-1\\ 2\leq I_{y_{i}}\leq M_{y}-1\\ 2\leq I_{z_{i}}\leq M_{z}-1. \end{array}$$ (22)

**Fig. 6:**
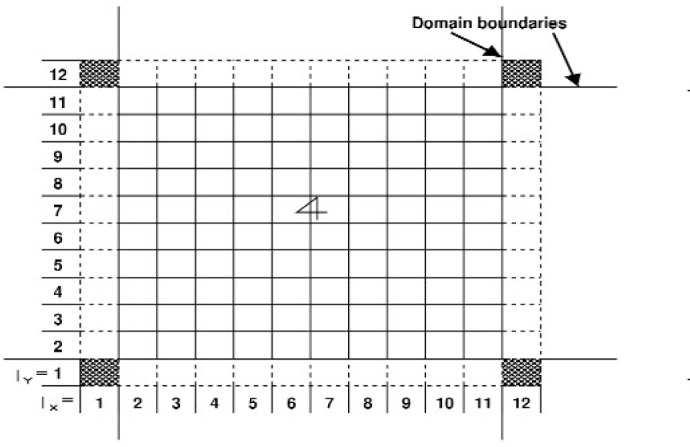
Enlargement of the central region of Figure 5. Link-cell periodic boundaries become processor domain boundaries. Dashed lines correspond to ghost cells.

In Fig. 6 we show the central processor from Fig. 5 again, with its “own” and “ghost” cells renumbered according to the above. Using these scale factors, it is straightforward to identify which particles need to be passed in all 4 (or 6) directions. For example, particles whose $I_{x_{i}}$ value is 2 are left edge particles and need to be passed to the processor to the left; particles whose $I_{x_{i}}$ value is 11 (*M_x_* − 1) are right edge particles and need to be passed to the processor on the right. It is important to note that particles in “ghost” cells are included in subsequent swaps, so for example particles whose $I_{y_{i}}$ value is 2 are passed down, and that includes particles in the “ghost” cells with $I_{y_{i}}=1$ and 12, and particles whose $I_{y_{i}}$ value is 1 are passed up. This is the way the particles in corner cells are made available to adjacent processors. As processor 4 communicates information about particles in its edge cells with *I_x_* = 11 to processor 5, processor 5 in turn communicates information about particles in its left edge cells to processor 4, which become the right edge ghost cells on processor 4. So after swapping with processors to its left, right, north, and south, the complete “extended volume” exists on processor 4, and this can be followed by the link-cell list construction (*I_x_* = {1,12}, *I_y_* = {1,12}) and computation of forces (for particles owned by this processor, which are those in cells with *I_x_* = {2,11}, *I_y_* = {2,11}).

Now consider Fig. 5 again, and imagine calculating the forces using a single processor and the link-cell algorithm, and subdividing the simulation box into 30 cells in *X* and *Y*. The force calculation on particles in cells with *I_x_*, *I_y_* = {11,20} in Fig. 5 would be calculated exactly the same way as the particles owned by processor 4 in Fig. 6, for which *I_x_*, *I_y_* = {2,11}. This is the essence of the parallel link-cell method.

**Fig. 7:**
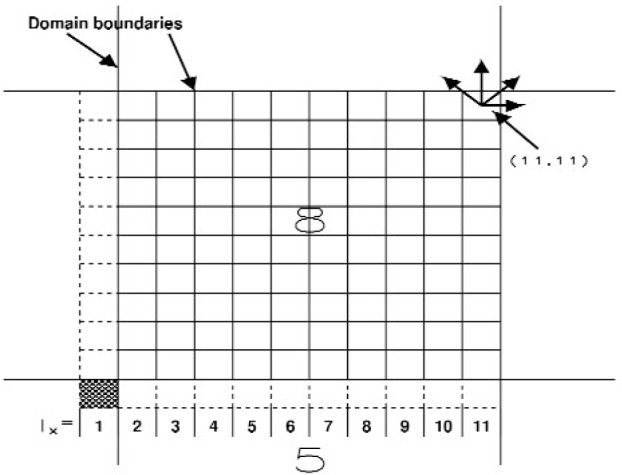
A 2-D example of the parallel link-cell algorithm showing a processor containing cells on the edge of the simulation box.

Similar conditions apply for the other processors, except for processors containing cells on the edge of the simulation box, such as processor 8 in Fig. 7. Cells interior to the processor, for which *I_x_*, *I_y_* are {2,10} are just like the cells on processor 4. At issue are the cells for which *I_x_* = 11 and those for which *I_y_* = 11, i.e, edge cells on the processor which are also edge cells for the whole simulation box (Fig. 5). But the right edge ghost cells (*I_y_* = 12) for processor 8 are *I_x_* = 1 cells for processor 6 and would be sent to processor 8 during the swap between these two processors (8 is the processor to the west (−*x* direction) of 6 and 6 is the processor to the east (+*y* direction) of 8). Similarly, processors 2 and 8 pair up to create the *I_y_* = 12 ghost cells on 8. The net result of this is that the force calculation on particles in the domain of processor 8 will be calculated exactly the same way as the force on particles in the cells with *I_x_* = {21,30}, *I_y_* = {21,30} in a sequential simulation of the whole box with 30 cells in *X* and *Y*. Similar conditions pertain to other processors containing cells on the edge of the simulation box.

**Fig. 8:**
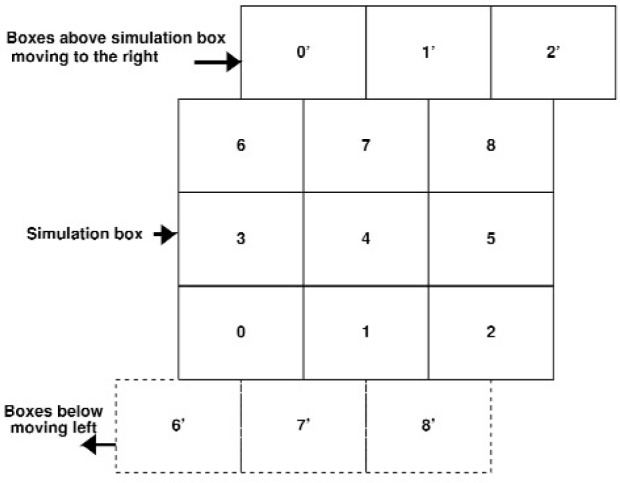
A nine processor 2-D domain decomposition and neighboring layers resulting from application of an applied strain consistent with the Lees-Edwards boundary condition.

**Fig. 9:**
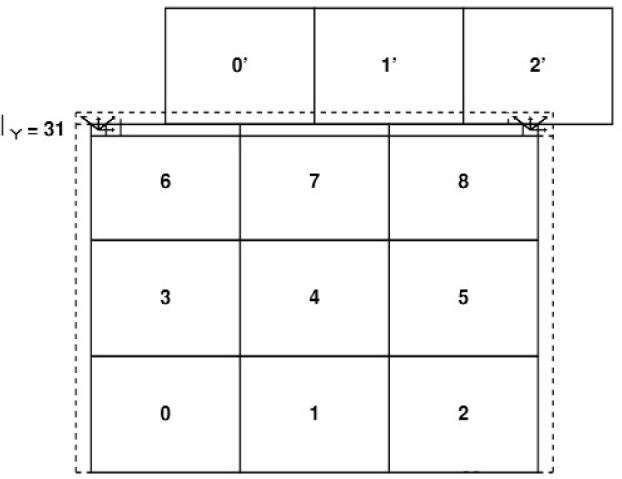
A more detailed nine processor 2-D domain decomposition including shear.

One point that was skipped in the above discussion is the treatment of the shear boundary conditions. In Fig. 8 we show the Fig. 5 simulation box again, and three boxes above the simulation box, moving to the right, as well as three boxes below the simulation box, moving to the left. 0′, 1′, and 2′ are images of 0, 1, and 2 which have moved to the right because of the shear. 6′, 7′, and 8′ have moved left. In Fig. 9 we redraw Fig. 8, showing the sheared upper boundary and the extended volume we have to build prior to computing forces. Cells that must be considered for edge cells (2,31) and (31,31) are shown with arrows. Note that because of Newton’s third law, the extended volume we need includes left, right, and up layers, but not down (*I_y_* = 1). Also care must be taken to include the shear shown in the figure. Subroutine EXTVOL handles this by forming the *Y* “ghost” layer before *X* (for 3D, the order is *Z*, *Y*, *X*). The *I_y_* = 32 layer is formed by processors 0, 1, and 2 sending their *I_y_* = 2 cells to processors 6, 7, and 8 respectively, and adding the simulator box distance in *Y*. In addition, movement to the right coming from the shear is computed from

r3(1,k)=r3(1,k)+strain10−ANINT(tempx/rmax(1))rmax(1) (23)

where *rmax*(1) is the simulation box dimension in *X* and

$$\begin{array}{l} tempx=r3(1,k)+strain10\\ strain10=rmax(1)*strain. \end{array}$$ (24)

Now subroutine LEBC is called to relocate particle properties to the processor that needs the information. This is done using the same technique as in MOVPAR, but care must be taken to keep track of the relocations so they can be reversed in the THIRDLAW transfer of forces back to their home processor. With these maneuvers, the Lees-edwards boundary condition is accomplished in our parallel program. Basically the program implements particles leaving at the bottom of the simulation box and entering at the top “ghost” layer (the mirror image) but with its *X* coordinate shifted to account for the strain.

## 5. Results and Discussion

**Fig. 10:**
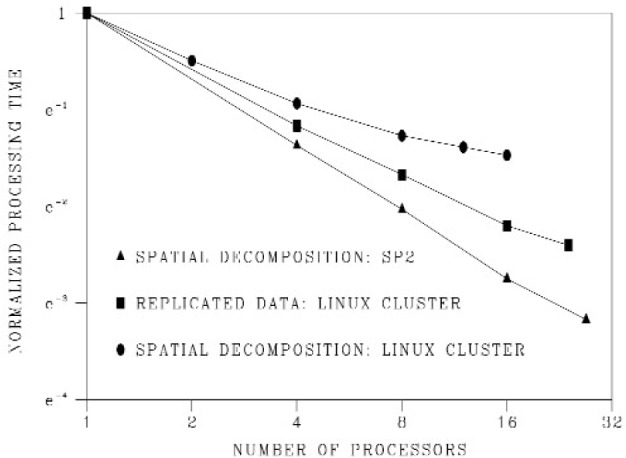
Logarithm (base e) of normalized CPU time (seconds) versus number of processors. The performance of the replicated data version degrades much more quickly than the spatial decomposition version of the same code.

Figure 10 shows the performance of our codes on two distributed memory architectures. In the figure we plot normalized processing time, which is the ratio of the time to complete a benchmark run on multiple processors divided by the time to compute a benchmark run on a single processor.

For the replicated data version of our code, the best we could do was a factor of 4.3 improvement on 16 processors on a Linux cluster with Myrinet. In comparison, the spatial decomposition version of the code, running on the same Linux cluster showed a greatly enhanced performance (a factor of 10.5 on 16 processors). The best results, for the spatial decomposition version, show a speed up of a factor of 24 on 27 200MHz Power3 processors on an IBM SP2, a distributed memory cluster, but with a high-speed interconnect which allows it to approach the scalability of a shared memory machine in many cases.

Our spatial decomposition code has proven effective in a shared memory environment [14] as well, where the speedups are a factor of 29 on 32 processors of an SGI Origin 3000 system and a factor of 50 on 64 processors of the same system. In contrast, for the replicated data parallelization, speedups are a factor of 17.5 on 24 processors of an SGI Origin 3000 [14]. Clearly, communication costs quickly become prohibitive for replicated data parallelizations on distributed memory architectures. Scaling to a very large number of processors is poor even in the shared memory environment, and it makes the replicated data approach almost unusable on distributed memory machines including those with high-speed interconnects like the IBM SP2 cluster.

## 6. Summary

In adopting a spatial decomposition approach, we found a significant improvment in performance of our codes despite the additional complications of communicating the random forces^3^, implementation of the Lees-Edwards boundary condition, and accounting for objects that can extend over many processor domains. Clearly, the main bottleneck of such an approach is the message passing between processors. As such technologies improve, we expect corresponding improvements in the computional performance of our algorithms.

Speedups like this on parallel architecture computers also allow us to systematically explore regions of parameter space (e.g., different solid fractions, broader particle size and shape distributions and other boundary conditions) that would be prohibitive on single processor computers. We also note for the record that this technique has proven effective in a shared memory environment [14] where the speedups were a factor of 29 on 32 processors of an SGI Origin 3000 system and a factor of 50 on 64 processors.

## Footnotes

1 Certain commercial equipment, instruments, or materials are identified in this paper to foster understanding. Such identification does not imply endorsement by the National Institute of Standards and Technology, nor does it imply that the materials or equipment identified are necessarily the best available for the purpose.

2 See Plimpton [16] for excellent discussions of all fast parallel algorithms.

3 The random force in the DPD formalism of particle *i* on particle *j* has to be equal and opposite of the force of particle *j* on particle *i*.
